# Elastic Stable Intramedullary Nailing and Temporary External Fixation for the Treatment of Unstable Femoral Shaft Fractures in Children Aged 5-11 Years Old: A Retrospective Study of 28 Cases

**DOI:** 10.3389/fped.2022.914834

**Published:** 2022-06-30

**Authors:** Yunan Lu, Federico Canavese, Ran Lin, Jinchen Chen, Yibin Chen, Yuling Huang, Shunyou Chen

**Affiliations:** ^1^Department of Pediatric Orthopedics, Fuzhou Second Hospital Affiliated to Xiamen University, The Third Clinical Medicine College of Fujian Medical University, Fuzhou, China; ^2^Department of Pediatric Orthopedic Surgery, Jeanne de Flandre Hospital, Lille University Centre, Lille, France; ^3^Fujian Provincial Clinical Medical Research Center for First Aid and Rehabilitation in Orthopedic Trauma, Fuzhou, China

**Keywords:** elastic stable intramedullary nailing, external fixator, pediatric femoral shaft fracture, diaphysis, unstable

## Abstract

**Purpose:**

Unstable femoral shaft fractures (UFSFs) in children aged 5–11 years remain challenging due to their intrinsic instability. The aim of this study was to evaluate the clinical and radiographic outcomes of UFSF in children aged 5 to 11 years managed by the combined use of ESIN and temporary EF.

**Methods:**

Children with UFSF (long oblique and comminuted) treated by ESIN and temporary EF were retrospectively reviewed. Sex, age at injury, side involved, type of fracture, presence or absence of associated lesions or neurovascular complications, type of treatment, time from trauma to surgery, duration of surgery, radiation exposure and length of postoperative immobilization were collected from the medical charts. Radiological and functional outcomes were evaluated according to Beaty's and Flynn's criteria, respectively.

**Results:**

A total of 28 consecutive patients with closed or open (Gustilo type I or II) UFSF were reviewed (18 boys and 10 girls). The mean age at injury was 8.7 ± 1.6 years (range, 5–11); the average weight was 38.1 ± 7.6 kg (range, 26–55). The mean hospital stay was 3.7 ± 1.4 days (range, 2–7), and the mean time to EF and ESIN removal was 6.5 ± 1.1 weeks (range, 4–8) and 9.4 ± 1.6 months (range, 6–12), respectively. Twenty-seven out of 28 patients had excellent radiographic outcomes according to Beaty's criteria, and 24/28 had excellent functional outcomes according to Flynn's criteria. Overall, 4 complications (14.3%) were recorded. No statistically significant correlation was found between complication rates and sex, age, weight or fracture characteristics (*P* < 0.05).

**Conclusions:**

The combined use of ESIN and temporary EF provides good clinical and radiological outcomes in children with UFSF aged between 5 and 11 years, with a reduced complication rate.

## Introduction

Pediatric diaphyseal femur fractures account for 1.6% of all pediatric fractures, with an annual incidence of 19 per 100,000 children (1). The treatment of pediatric diaphyseal femur fractures is relatively well codified with regard to indications based on the age and weight of the patient as well as the type of fracture. However, it remains controversial in regard to unstable (comminuted and long oblique) diaphyseal femur fractures occurring in children between 5 and 11 years of age (2). Indeed, the use of traction followed by spica cast immobilization and rigid intramedullary nails are often the treatments of choice in children <5 and >11 years of age (3, 4).

Elastic stable intramedullary nailing (ESIN) is particularly indicated for transverse fractures because of its simple procedure, no need for casting after surgery and early physiotherapy (5, 6). However, whether it should be indicated for severe comminuted or long oblique diaphyseal fractures of the femur remains unclear because of its inability to control the length and rotation of the fractured bone (7, 8).

External fixation (EF) plays a role in the management of unstable diaphyseal fractures, but a number of studies have reported significant complications, such as pin tract infections, malunion, loss of reduction and refracture (9, 10).

Only a limited number of studies have focused on the combined use of ESIN and EF. Ertürk et al. (11) and Atef and El Tantawy (12) reported the combined use of ESIN and EF in the management of open unstable tibial fractures in adults and adolescents. However, no studies have reported the results of such a fixation system in children aged 5 to 11 years with unstable femur fractures.

Our hypothesis is that unstable femur shaft fractures (UFSFs) in children aged 5 to 11 years can be treated by the combined use of ESIN and EF, with complication rates and clinical outcomes comparable to those of ESIN or EF alone.

The aim of this study was to retrospectively evaluate the clinical and radiographic outcomes of UFSF in children aged 5 to 11 years managed by the combined use of ESIN and EF.

## Materials and Methods

After securing approval from the Institutional Review Board (No. 2022006), a retrospective review of medical charts was performed to identify children and adolescents who presented at the Emergency Department (ED) of our Institution for femur shaft fracture from March 2016 to April 2020.

A total of 289 pediatric patients with femoral shaft fractures were admitted to our institution during the study period; patients were consecutively enrolled, and all fractures were managed at a single institution by the same surgical team.

Patients were admitted through the ED, and the following data were collected: sex, age at injury, side involved, type of fracture (transverse, oblique or comminuted), presence or absence of associated lesions or neurovascular complications and whether the fracture was closed or open. Additional data, such as type of treatment, time from trauma to surgery, duration of surgery, radiation exposure and length of postoperative immobilization, if any, were also collected from the charts.

The inclusion criteria were as follows: (1) confirmed diagnosis of UFSF (comminuted and long oblique); (2) closed or open Gustilo type I or II injury; (3) age between 5 and 11 years of age, regardless of body weight; (4) treatment by the combined use of ESIN and EF; (5) treatment within 2 weeks of the initial trauma; (6) complete clinical and radiographic data; and (7) follow-up more than 12 months.

The following exclusion criteria were applied: (1) fractures not involving the femur diaphysis (fractures located within 5 cm of the proximal or distal articular surface and intra-articular fractures); (2) transverse fracture; (3) pathological or open Gustilo type III fractures; (4) patients younger than 5 years of age and older than 11 years at the time of injury; (5) fractures treated with techniques other than the one reported in this study or patients treated at another institution; (6) incomplete clinical or radiographic data; and (7) follow-up less than 12 months.

**Figure 1:**
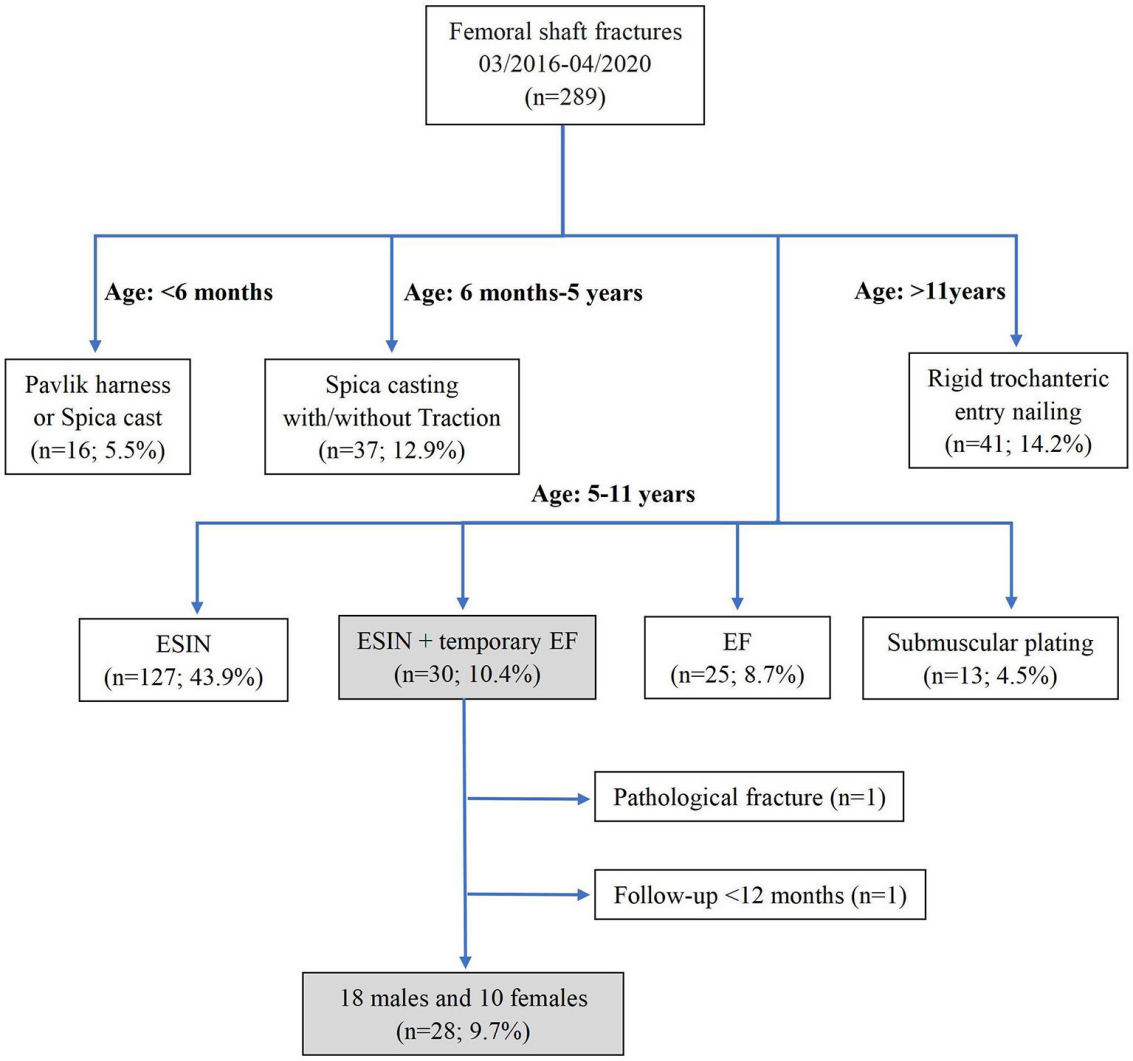
Flowchart of cases.

Twenty-eight out of 289 patients met the inclusion criteria and were analyzed in this study. There were 18 boys and 10 girls. Figure 1 summarizes the flow chart of the patients (Figure 1).

### Surgical Technique

**Figure 2:**
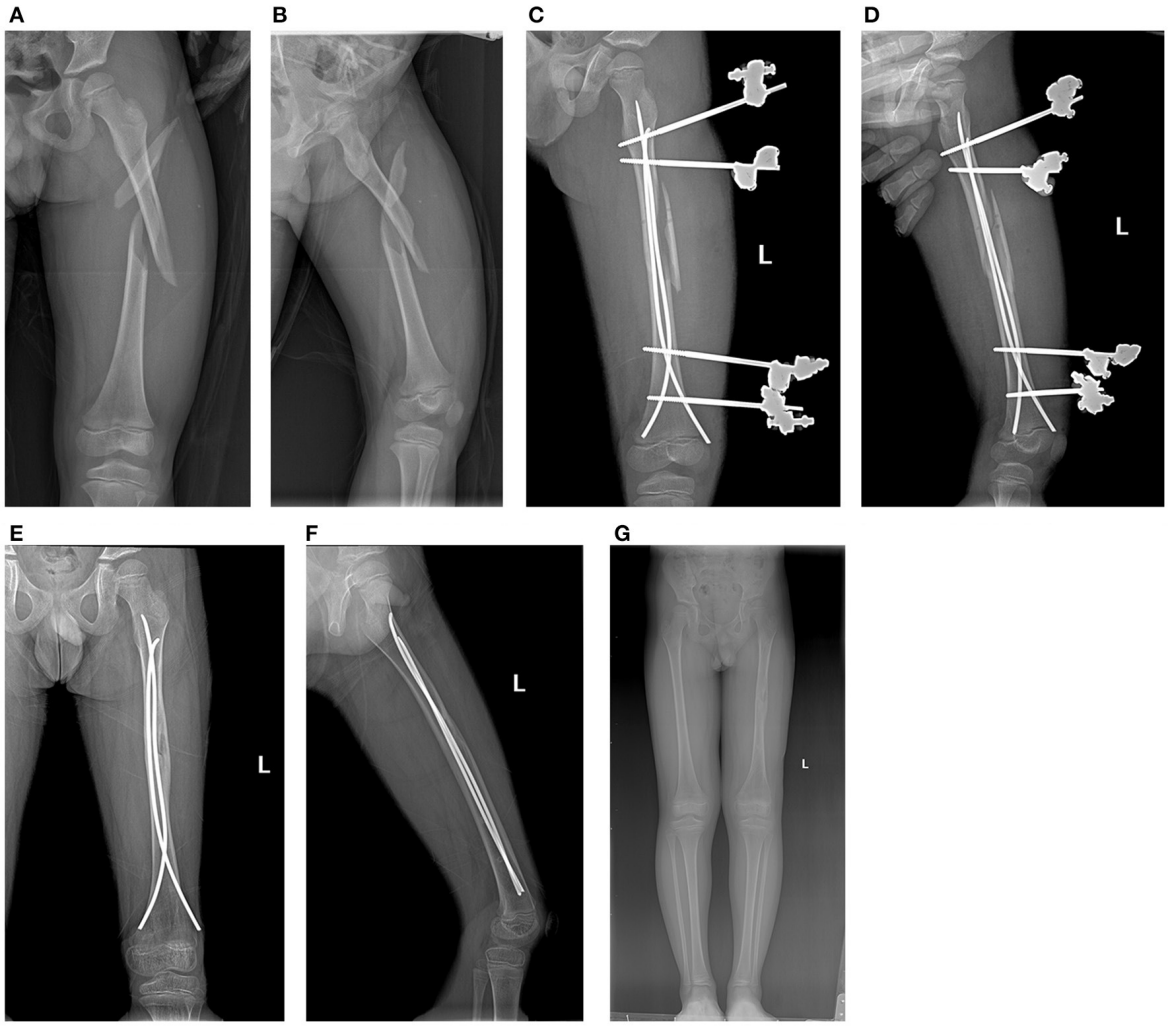
Case 5. **(A,B)** Comminuted type III femur fracture in an 8-year-old boy; **(C,D)** postoperative radiographs; **(E,F)** removal of EF; **(G)** final outcome.

**Figure 3:**
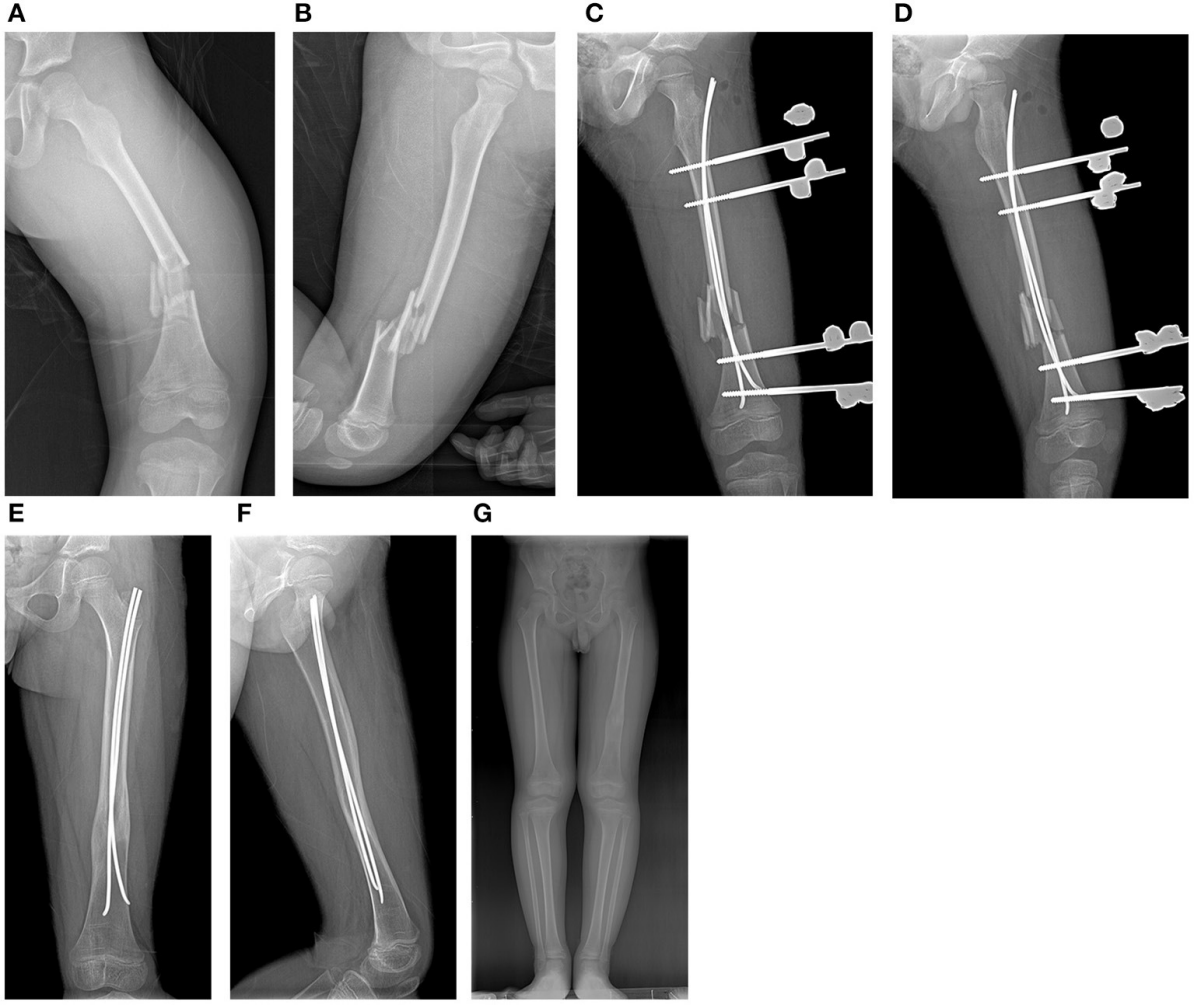
Case 10. **(A,B)** Comminuted type IV femur fracture in an 8-year-old boy; **(C,D)** postoperative radiographs; **(E,F)** removal of EF; **(G)** final outcome.

**Figure 4:**
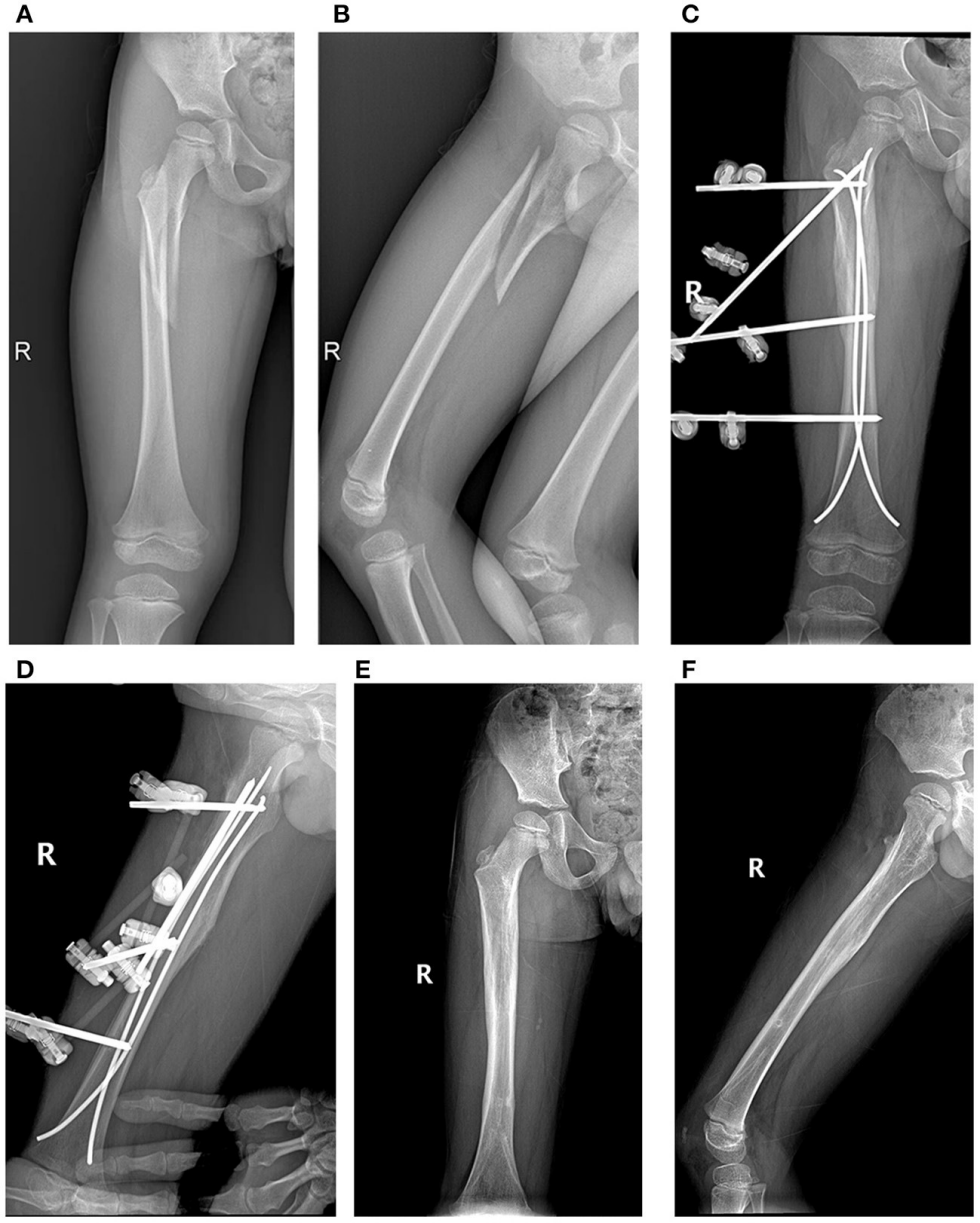
Case 18. **(A,B)** Long oblique proximal fracture of a 7-year-old boy; **(C,D)** postoperative radiographs; **(E,F)** final outcome.

All surgeries were performed on a radiolucent fracture table. All surgeries were performed by the same experienced pediatric orthopedic surgeon (S.C.). Two C-shaped titanium elastic nails (TEN; Synthes, Solothurn, Switzerland) were inserted retrograde when the fracture site was located in the proximal and middle third of the femur; one C- and one S-shaped elastic nail were inserted anterograde when the fracture was located in the distal third (Figures 2–4). Fracture reduction was achieved by traction and an external maneuver. The nail diameter was predetermined to fill the medullary canal with 35**–**40% of the narrowest diameter of the femur (8, 13). Once the two elastic nails were inserted, Hoffmann-II EF (SK-external fixators, Double, Xiamen, China) was applied to achieve the final reduction and to control the length and rotation of the fractured bone. Specifically, the foot and the patella were used to judge rotation, while the contralateral limb was used to evaluate the length and axis.

### Radiographic Evaluation

All patients underwent full-length antero-posterior (AP) and lateral radiographs of the injured femur. The femoral shaft was defined as the portion of the femur between the area just distal to the neck and the area immediately proximal to the supracondylar ridge (8).

Using the AP and lateral radiographs, fractures were classified as oblique (long or short), transverse, spiral or comminuted on the basis of the relationship between the fracture line and the axis of the femur shaft and the amount of fragmentation. Long oblique and long spiral fractures were defined, in accordance with the criteria of Sink et al. (14), as those in which the length of the obliquity was at least twice the size of the diameter of the diaphysis at the fracture site.

The magnitude of comminution was graded according to the Winquist and Hansen classification (15). This classification is based on the percentage of fragmentation of the femoral diaphysis width, and it allows for the identification of four categories: type 1: fragmentation <25%; type 2: fragmentation between 25 and 50%; type 3: fragmentation between 50 and 75%; type 4: fragmentation >75% or segmental. Fractures were also graded according to the AO/OTA system (16).

All measurements were performed using the Picture Archiving and Communication Systems (PACS; GE Healthcare, Chicago, IL). Two experienced pediatric orthopedic surgeons (YL and RL) measured all parameters independently, and the mean values were used for the statistical analysis.

### Clinical Evaluation and Follow-Up

No additional immobilization of the affected limb was required after surgery. Hip and knee functional exercises started on the first day post-surgery. Weight bearing was allowed as tolerated by the patient 3 to 5 days after the initial surgery. The EF was removed 4 to 8 weeks after the initial surgery based on the presence of callus formation. The elastic nails were removed 6 to 12 months after fracture consolidation.

All patients underwent regular clinical and radiologic follow-up in the outpatient clinic every 2 weeks for the first 2 months and at 2-month intervals thereafter. All patients were followed for at least 12 months (range, 18**–**30). The hip and knee range-of-motion (ROM) of the affected side were assessed at each follow-up visit and compared with those of the uninjured side. Beaty's criteria were used to evaluate the radiological outcome (17) (Table 1): malunion was defined as anterior bowing greater than 15°, or varus or valgus greater than 10° (18). Flynn's Titanium Elastic Nail (TEN) grading system was used to assess the clinical outcome (8) (Table 2).

**Table 1:** Beaty's criteria (radiological outcome).

	**Satisfactory**	**Poor**
Shortening	<1 cm	>1 cm
Lengthening	None	Present
Coronal angulation	<5°	>5°
Sagittal angulation	<10°	>10°

**Table 2:** Flynn's titanium elastic nails grading system (functional outcome).

	**Excellent**	**Satisfactory**	**Poor**
Limb length discrepancy	<1 cm	1–2 cm	>2 cm
Malalignment	<5°	5-10°	>10°
Pain	None	None	Present
Complication	None	Minor/resolved	Major/lasting

### Statistical Analysis

Categorical variables are expressed as frequencies and percentages. Quantitative data are expressed as the means, ranges and standard deviations (SD). Statistical analysis was performed using Fisher's exact test for categorical variables. Data were analyzed using the IBM SPSS statistical package version 22.0 (IBM Corporation, Armonk, NY, USA). The threshold for statistical significance was set to a *p* value less than 0.05.

## Results

A total of 28 consecutive patients with closed or Gustilo type I and II UFSF were reviewed (18 boys and 10 girls). The mean age at injury was 8.7 ± 1.6 years (range, 5**–**11); the average weight was 38.1 ± 7.6 kg (range, 26**–**55). Table 3 summarizes the demographics of the patients.

**Table 3:** Demographic of patients.

**Case**	**Gender**	**Age (ys)**	**Weight (Kg)**	**Mechanism of injury**	**Fracture**				
					**Type**	**Side**	**Location**	**Pattern**	**AO/OTA**
1	F	10	38	FH	Closed	R	Mid. 1/3rd	Comminuted-III	B1.1
2	M	8	32	TA	Closed	L	Dis. 1/3rd	Comminuted-III	B2.1
3	M	5	28	FH	Closed	L	Mid. 1/3rd	Comminuted-IV	C3.1
4	M	11	53	SI	Closed	L	Dis. 1/3rd	Comminuted-IV	C3.1
5	M	8	32	TA	Closed	L	Mid. 1/3rd	Comminuted-III	B1.1
6	M	11	45	SI	Closed	L	Mid. 1/3rd	Comminuted-III	B1.1
7	M	8	31	SI	Closed	R	Pro. 1/3rd	Comminuted-III	B2.1
8	M	8	43	TA	Closed	R	Pro. 1/3rd	Long oblique	A2.1
9	M	10	36	SI	Closed	R	Mid. 1/3rd	Comminuted-III	B1.1
10	M	8	41	FH	Closed	L	Dis. 1/3rd	Comminuted-IV	C3.1
11	F	8	45	TA	Closed	L	Dis. 1/3rd	Long oblique	A2.1
12	M	7	32	TA	Closed	R	Mid. 1/3rd	Comminuted-III	B1.1
13	M	9	35	FH	Closed	L	Mid. 1/3rd	Comminuted-III	B1.1
14	F	7	38	FH	Closed	L	Dis. 1/3rd	Long oblique	A2.1
15	M	10	52	TA	Closed	L	Mid. 1/3rd	Comminuted-III	B1.1
16	F	6	32	TA	Closed	R	Mid. 1/3rd	Comminuted-III	B2.1
17	M	8	33	FH	Closed	L	Mid. 1/3rd	Comminuted-IV	C3.1
18	M	7	29	SI	Closed	R	Pro. 1/3rd	Long oblique	A2.1
19	M	9	34	SI	Closed	R	Mid. 1/3rd	Comminuted-IV	C3.1
20	F	10	42	TA	Gustilo-II	R	Mid. 1/3rd	Comminuted-III	B2.1
21	M	11	40	TA	Closed	R	Dis. 1/3rd	Comminuted-III	B2.1
22	M	9	42	SI	Closed	L	Dis. 1/3rd	Long oblique	A2.1
23	F	10	45	TA	Closed	R	Dis. 1/3rd	Comminuted-IV	C3.1
24	F	11	55	FH	Closed	R	Pro. 1/3rd	Comminuted-III	B2.1
25	M	10	45	TA	Closed	R	Mid. 1/3rd	Comminuted-IV	C3.1
26	F	7	26	FH	Gustilo-I	L	Mid. 1/3rd	Long oblique	A2.1
27	F	8	30	SI	Closed	L	Mid. 1/3rd	Comminuted-IV	C2.1
28	F	9	34	FH	Closed	R	Pro. 1/3rd	Long oblique	A2.1

*M, male; F, female; L, left; R, right; Pro. 1/3^rd^, Proximal third; Mid.1/3^rd^, Middle 3^rd^; Dis. 3^rd^, Distal 3^rd^; TA, Traffic accident; FH, Falling from height; SI, Sports injury*.

The mechanism of injury was traffic accidents in 11 patients (39.3%), falls from a height in 9 patients (32.1%), and sports injuries in 8 patients (28.6%).

Seven fractures (25.0%) were classified as long oblique or A2.1 according to the AO/OTA classification, 13 (46.4%) as Winquist and Hansen grade III comminuted fractures or B1.1/B2.1 according to the AO/OTA classification, and 8 (28.6%) as grade IV comminuted fractures or C2.1/C3.1 according to the AO/OTA classification.

The fracture was located in the middle third of the femur diaphysis in 15 patients (53.6%), the proximal third in 5 patients (17.9%), and the distal third in 8 patients (28.6%).

The mean time of the surgery was 64.8 ± 10.2 min (range, 50–90). The mean duration of hospitalization was 3.7 ± 1.4 days (range, 2**–**7). The mean follow-up time was 21.8 ± 2.7 months (range, 18**–**30).

The EF was removed at an average of 6.5 ± 1.1 weeks after the index surgery (range, 4–8), while the elastic nails were removed at an average of 9.4 ± 1.6 months from the initial surgical procedure (range, 6**–**12).

All fractures healed, without delayed union, malunion or refracture. According to Beaty's radiological outcome criteria (Table 2), 27 out of 28 patients had excellent radiological outcomes (96.4%).

According to Flynn's TEN outcome grading system (Table 3), 24 out of 28 patients (85.7%) had excellent results, and 4 patients (14.3%) had satisfactory results; no poor results were observed. Two cases of pin tract infections were recorded that healed without complications after removal of the pin. Temporary stiffness of the knee joint was observed in one patient but it returned to normal after removal of the EF and subsequent rehabilitation. One patient had a lower limb discrepancy of 13 mm at the last follow-up visit. Table 4 summarizes the clinical outcomes and complications (Table 4).

**Table 4:** Outcome.

**Case**	**Hospital stay (days)**	**Follow-up (months)**	**EF removal (weeks)**	**ESIN removal (months)**	**Beaty's criteria**	**Flynn's scale**	**Complications**
1	3	21	6	9	Excellent	Excellent	-
2	5	26	8	7	Excellent	Excellent	-
3	4	24	6	8	Excellent	Excellent	-
4	3	22	8	11	Excellent	Satisfactory	Pin tract infection
5	3	18	7	9	Excellent	Excellent	-
6	6	21	8	12	Excellent	Satisfactory	Knee joint stiffness
7	2	26	5	8	Excellent	Excellent	-
8	2	22	6	12	Excellent	Excellent	-
9	4	23	6	8	Excellent	Excellent	-
10	4	19	8	10	Excellent	Excellent	-
11	3	20	6	9	Excellent	Excellent	-
12	2	18	5	8	Excellent	Excellent	-
13	4	22	6	11	Excellent	Excellent	-
14	3	24	6	9	Excellent	Excellent	-
15	4	22	8	8	Excellent	Excellent	-
16	3	30	6	9	Excellent	Excellent	-
17	3	19	8	8	Excellent	Excellent	-
18	2	23	6	6	Excellent	Excellent	-
19	4	21	6	12	Excellent	Excellent	-
20	6	18	8	10	Excellent	Excellent	-
21	2	20	4	8	Poor	Satisfactory	Overgrowth 13 mm
22	2	24	6	9	Excellent	Excellent	-
23	5	22	6	11	Excellent	Excellent	-
24	3	20	6	12	Excellent	Excellent	-
25	3	23	7	9	Excellent	Excellent	-
26	7	20	7	11	Excellent	Satisfactory	Pin tract infection
27	7	21	8	9	Excellent	Excellent	-
28	4	20	6	10	Excellent	Excellent	-

Table 5 outlines the complications according to sex, age, weight and fracture type, location and pattern. No statistically significant correlation was found between the complication rate and any of these factors (*P* < 0.05) (Table 5).

**Table 5:** Complications according to gender, age, weight and fracture characteristics.

	**No complications (*n =* 24)**	**Complications (*n =* 4)**	***P*-value**
Gender			
Male	15 (83.3%)	3(16.7%)	1.000
Female	9 (90%)	1(10%)	
Age			
<9 years	13 (92.9%)	1 (7.1%)	0.596
≥9 years	11 (78.6%)	3 (21.4%)	
Weight			
<35 Kg	11 (91.7%)	1 (8.3%)	0.613
≥35 Kg	13 (81.3%)	3 (18.8%)	
Fracture type			
Closed	23 (88.5%)	3 (11.5%)	0.270
Open	1 (50%)	1 (50%)	
Fracture location			
Pro. 1/3^rd^	4 (80.0%)	1 (20.0%)	0.451
Mid. 1/3^rd^	14 (93.3%)	1 (6.7%)	
Dis. 1/3^rd^	6 (75%)	2 (25%)	
Fracture pattern			
Long oblique	6 (85.7%)	1 (14.3%)	0.983
Comminuted-III	11 (84.6%)	2 (15.4%)	
Comminuted-IV	7 (87.5%)	1 (12.5%)	

## Discussion

Our study shows that UFSF in children aged 5 to 11 years of age can be treated by ESIN and temporary EF; functional and radiological outcomes are generally good to excellent, and the complication rate is comparable to ESIN or EF alone (3, 8, 9, 14); in particular, only one case of lower limb length discrepancy was detected among patients treated by the reported technique.

Our results are in agreement with reports that applied such a technique to adolescents and adults with unstable diaphyseal fractures of the tibia (12); in particular, Ertürk et al. and Atef and El Tantawy reported that the procedure is minimally invasive, avoids malunion and allows bone healing (11, 12).

The management of UFSF in children aged 5 to 11 years remains controversial. ESIN is the most common treatment method in this age group, mostly for children with transverse fracture and with a body weight less than 50 kg, because the technique is minimally invasive with a low complication rate (7, 8, 13). However, Narayanan et al. pointed out that while ESIN is a versatile technique, fractures with more than 25% fragmentation are over four times more likely to result in a loss of reduction and to require reoperation (19). Sink et al. in their series of 21 patients with UFSF, reported an overall complication rate of 57%, and the majority of their patients (71%; *n* = 15) were managed by ESIN (14). The authors concluded that methods other than ESIN should be used in children with UFSF (8, 14, 19, 20). Similarly, the AAOS suggested that ESIN should be reserved for transverse and short oblique femoral shaft fractures but not applied to UFSF since it is associated with a higher rate of complications (2). Other techniques, such as rigid intramedullary nailing and submuscular plating, have limitations. In particular, rigid nailing may cause growth disturbance and avascular necrosis of the proximal femur epiphysis, while submuscular plating carries a high risk of refracture because of stress shielding and valgus deformities, especially in distal third fractures (21, 22).

EF is a quick and minimally invasive technique for the management of UFSF in children younger than 11 years of age (23), although the rate of pin site infection and refracture following removal is not negligible (9, 10).

In children with UFSF, we used ESIN and temporary EF. Specifically, ESIN can correct the alignment, while EF controls the rotation and limb length, prevents secondary displacement, and allows for early weight-bearing. In our series, the EF was removed on average at 6.5 weeks after the initial surgery, when the bone callus was sufficiently formed. From this point on, the presence of the elastic nails allows the forces to be distributed between the bone and the hardware, allowing the fracture to consolidate permanently. We did not observe any cases of malunion, and the comminuted fragments healed in all cases.

Excellent radiological outcomes were observed in 96.4% of patients (27/28), with a low rate of complications, early motion (first postoperative day), rapid weight bearing (between the 3^rd^ and 5^th^ postoperative days) and a short hospital stay (3.7 days on average).

In our opinion, the use of ESIN and temporary EF has the following advantages:

1) The system maintains alignment and length, controls rotation and does not require postoperative immobilization. Particularly, it allows early weight bearing and mobilization, and it reduces the potential discomfort caused by plaster or braces, as pointed out by Flynn et al. (8);
2) The system is semirigid and allows micromotion at the fracture site, which is beneficial to bone healing. In addition, it does not completely fill the medullary cavity, and endosteal callus formation is not inhibited (13);
3) The EF can be easily modulated and adapted to the fracture pattern (Figure 3). In addition, the use of carbon components allows the bone anatomy to be fully appreciated on plain radiographs;
4) The system can also be used in patients weighing more than 50 kg; Moroz et al. (24) and Canavese et al. (25) reported that there is an increased risk of complications with ESIN in patients over 50 kg; in our study, however, the system could be used in this subgroup of patients without any complications.

Despite the advantages mentioned above, the addition of temporary EF may increase the operation time, compared with simple ESIN fixation, and there may be some difficulties in the placement of Schanz's pins after ESIN have implanted. The combined system is also at risk of pin tract infection and joint stiffness, as reported for the use of EF alone (9, 10).

Pin tract infection is relatively common in patients treated by EF, although we encountered only two cases of such complications in our series of patients; local treatment of the infection and removal of the EF allowed healing without sequelae. Interestingly, the overall infection rate in our series was lower than that in previous reports using EF alone for the treatment of femoral shaft fractures (9, 10). This is probably related to the fact that the EF is removed early (mean: 6.5 weeks), while the elastic nails decrease the weight-bearing forces at the pin-bone interface. Moroni et al. found that callus bending stiffness increases between the 3^rd^ and the 7^th^ week post-fracture, and Schanz-pin loosening starts after the 8^th^ week (26). We recommend removal of the EF once the callus has formed sufficiently and before pin loosening starts.

Joint stiffness is another complication of EF that cannot be overlooked. In our study, only one patient developed knee joint stiffness. However, active knee exercises were partially hampered by the size of the quadriceps femoris, which, given its anatomic location envelops the femoral diaphysis, increasing friction between the muscle and Schanz's pin (27). However, the EF was routinely removed at 4–8 weeks postoperatively, and active exercises were encouraged to recover full knee joint ROM.

There was one case of a lower limb length discrepancy of 13 mm. Lower limb overgrowth after femur fractures can be observed, although its etiology is not clear. Park et al. reported that unstable fractures and large periosteal dissection may lead to overgrowth (28). On the other hand, we did not record any deformity following surgical treatment of UFSF by ESIN, which is in contrast with previous reports (8, 14, 19).

All of the complications mentioned above are relatively common and are inherent to EF and UFSF. We found that our rates of these complications were much lower than those reported in previous studies on the use of EF or ESIN alone (8–10, 14, 19), and, in most cases, they were minor and did not require a second operation. Furthermore, our data showed that such complications were independent of age, weight, fracture pattern and location (*P* < 0.05).

This technique is valid for both Gustilo type I and II open fractures. In particular, it is important to note that the reported technique was first used for Gustilo type I to IIIA open tibial fractures in adults with good functional outcomes and good soft tissue coverage compared to plate or interlocking intramedullary nailing (11). ESIN does not increase the rate of infection in cases with open fractures as long as urgent and thorough wound debridement and effective use of antibiotics are performed (11, 12).

We encountered some limitations in the analysis of our results. This was a retrospective review of prospectively enrolled patients, with a relatively low number of patients. Even so, to the best of our knowledge, this is the first study that has evaluated the clinical and radiological outcomes of UFSF in children between 5 and 11 years of age managed by the combined use of ESIN and temporary EF. The retrospective nature of our study is prone to selection and observational biases, and randomization was not possible; however, the patients were consecutive, all came from a single institution, and the total number of cases was higher than those in other published studies (29, 30). Finally, the retrospective nature of our study also limited the level of evidence for the findings, as our series lacks a control group treated with other surgical techniques. Multicenter, large-sample studies with long follow-up times are now needed for more robust results.

## Conclusion

The combined use of ESIN and temporary EF provides good clinical and radiological outcomes in children with UFSF aged between 5 and 11 years, with a reduced complication rate.

## Data Availability Statement

The raw data supporting the conclusions of this article will be made available by the authors, without undue reservation.

## Ethics Statement

The studies involving human participants were reviewed and approved by Institutional Review Board (No. 2022006). Written informed consent was obtained from the minor(s)' legal guardian/next of kin for the publication of any potentially identifiable images or data included in this article.

## Author Contributions

YL and FC: design of the study, manuscript preparation, statistical analysis, and revision. YC and RL: drafted the manuscript. YH and JC: statistical analysis and revision of the manuscript. SC: design of the study and surgery and general supervision of the research group. All authors read and approved the final manuscript.

## Funding

This work was supported by Fujian Provincial Clinical Medical Research Center for First Aid and Rehabilitation in Orthopedic Trauma (2020Y2014).

## Conflict of Interest

The authors declare that the research was conducted in the absence of any commercial or financial relationships that could be construed as a potential conflict of interest.

## Publisher's Note

All claims expressed in this article are solely those of the authors and do not necessarily represent those of their affiliated organizations, or those of the publisher, the editors and the reviewers. Any product that may be evaluated in this article, or claim that may be made by its manufacturer, is not guaranteed or endorsed by the publisher.
